# Hsa_circ_0003220 Drives Chemoresistance of Human NSCLC Cells by Modulating miR-489-3p/IGF1

**DOI:** 10.1155/2023/8845152

**Published:** 2023-06-16

**Authors:** Shaofeng Xia, Chenliang Wang

**Affiliations:** ^1^Department of Thoracic Surgery, The First People's Hospital of Jiujiang City, Jiujiang, Jiangxi, China; ^2^Department of Pathology, The First People's Hospital of Jiujiang City, Jiujiang, Jiangxi, China

## Abstract

Circular RNAs (circRNAs) have been shown to have critical roles in developing cancer and treatment resistance in an increasing body of research. The aim was to look into the functions and processes of hsa_circ_0003220 in the non-small cell lung cancer (NSCLC) chemoresistance. The NSCLC cell lines H460 and A549 were employed in present work. hsa_circ_0003220, miR-489-3p, and insulin-like growth factors (IGF1) mRNA levels were assessed with a quantitative real time polymerase chain reaction (qRT-PCR). The cisplatin, docetaxel, and paclitaxel (PTX) resistances were determined using 3-(4,5-dimethylthiazol-2-yl)-2,5-diphenyltetrazolium bromide assay, and the enzyme linked immunosorbent assay (ELISA) test measured IGF1 expression. In order to corroborate the miR-489-3p relation with hsa_circ_0003220 or IGF1, a dual-luciferase reporter method was applied. The level of hsa_circ_0003220 was raised in cells and tissues from PTX-resistant (PR) NSCLC. In PR NSCLC cells, hsa_circ_0003220 knockdown reduced chemoresistance. For the purpose of the mechanism study, hsa_circ_0003220 knockdown substantially reduced IGF1 expression via miR-489-3p sponging, reducing chemoresistance in PR NSCLC cells. By controlling the miR-489-3p/IGF1 axis, hsa_circ_0003220 knockdown helped NSCLC overcome chemoresistance, suggesting a potential circRNA-targeted therapy for the disease.

## 1. Introduction

With an occupancy rate of over 80%, non-small cell lung cancer (NSCLC) is the most frequent lung cancer [1, 2]. NSCLC is still the top cause of cancer mortality, despite the substantial progress that has been made in detecting and treating the disease [3]. Surgical procedures, radiation therapy, chemotherapy, and immunotherapy are the current modes of treatment for NSCLC [4]. Paclitaxel (PTX) is a medication used to treat different forms of cancer, such as NSCLC [5]. Nevertheless, the development of resistance to treatment drugs is a significant challenge for chemotherapy [6]. Therefore, it is essential to find innovative ways to get around the fact that NSCLC patients have developed a resistance to PTX.

Circular RNAs, also known as circRNAs, are a kind of noncoding RNA that can be identified by their closed-loop structures and the absence of both 3′ poly(A) tails and 5′ caps [7]. circRNAs are produced mostly from exons or introns, with exonic circRNAs being primarily expressed in the cytoplasm and exhibiting higher levels of stability compared with linearity RNAs [8]. In recent years, circRNA plays a significant role in tumor. For example, circRNA_101237 promotes NSCLC progression via the miRNA-490-3p/MAPK1 axis [9]. Serum extracellular vesicle-derived circHIPK3 and circSMARCA5 are two novel diagnostic biomarkers for glioblastoma multiforme [10]. CircRNA_100565 contributes to cisplatin (DDP) resistance of NSCLC cells by regulating proliferation, apoptosis, and autophagy via miR-337-3p/ADAM28 axis [11]. It has been clear that an increasing number of circRNAs are engaged in controlling gene expressions by sponging miRNAs [12, 13]. This discovery has been made in the last few years. Then, an increasing number of studies demonstrate that aberrant amounts of circRNAs are substantially implicated in the carcinogenesis and development of many cancers, such as NSCLC [14, 15]. An increasing pile of studies has shown that circRNAs may operate as oncogenes or cancer promoters in a variety of malignancies by binding to miRNAs in a competitive fashion [16, 17]. Despite the fact that a number of rising circRNAs have been found to have a dysregulated level in NSCLC, the role of these circRNAs and the associated molecular processes in the development of NSCLC have remained largely unknown.

Hsa_circ_0003220 is a recently discovered circular RNA with a length of 103 nucleotides and was produced by the reverse splicing of TMCO7 mRNAs. In this study, our objective was to evaluate the miR-489-3p and hsa_circ_0003220 expression in PTX-resistant (PR) NSCLC cells, as well as their potential roles. Analyses were conducted on the functional aspects of chemoresistance and tumor development in vivo. The connection between hsa_circ_0003220 and miR-489-3p was verified using mechanical means, and insulin-like growth factors (IGF1), which was further validated as one of miR-489-3p-target 3p's genes, was also validated. Based on these findings, hsa_circ_0003220, miR-489-3p, and IGF1 may provide potential therapeutic targets for treating chemoresistant NSCLC.

## 2. Materials and Methods

### 2.1. Tissues Acquisition

There were 132 cases of NSCLC for which tissues were collected with their matched noncancerous counterparts. At Jiujiang's First People's Hospital, 146 NSCLC tissue samples were taken from patients with written informed consent. Based on how NSCLC patients responded to PTX, the samples were divided into two groups: the treatment-resistant group (*n* = 64) and the treatment-sensitive group (*n* = 82), respectively. Until usage, the samples were kept at −80°C. The First People's Hospital of Jiujiang's Ethics Committee approved the project.

### 2.2. Cell Culture

293T, H460, A549, and primary human bronchial epithelial (HBE) cells were procured from Procell (China). H460 and A549 NSCLC cells resistant to PTX were created by subjecting the parent cells to higher dosages of PTX (SP8020; Solarbio, Beijing, China). The H460, A549, and HBE was cultured at 37°C with 5% CO_2_ in 10% fetal bovine serum (FBS) and 1% penicillin–streptomycin supplemented RPMI1640 media (Invitrogen, Carlsbad, CA, USA). The 293T was cultured at 37°C with 5% CO_2_ in 10% FBS and 1% penicillin–streptomycin supplemented dulbecco's modified eagle medium (DMEM) media (Invitrogen, Carlsbad, CA, USA). Additionally, media was added with 5 nM PTX (Solarbio) to keep the H460/PTX and A549/PTX cells resistant.

### 2.3. Cell Transfection

The IGF1 overexpression vector (IGF1), empty pcDNA, si-NC, hsa_circ_0003220 small interfering RNA, miR-489-3p mimics (miR-489-3p), and miR-NC, miR-489-3p inhibitors (anti-miR-489-3p), and anti-miR-NC were all created. After plating H460/PTX and A549/PTX cells (2 × 10^4^ cells/well) in 6-well plates for 24 hours, Lipofectamine 2000 (Invitrogen) was used to transfect the H460/PTX and A549/PTX cells with vectors (2 *μ*g) or suitable synthetic oligonucleotides (50 nM) according to the package recommendations.

### 2.4. qRT-PCR Assay

Total RNA was isolated using the RNeasy Mini kit (Qiagen, CA, USA) and quantified utilizing Thermo Fisher NanoDrop 2000c spectrophotometer (USA). Either the M-MLV Reverse Transcriptase Kit (Promega, Fitchburg, WI, USA) or the TaqMan MicroRNA Reverse Transcription Kit (Applied Biosystems, USA) was subsequently used to construct cDNAs. The quantitative real time polymerase chain reaction (qRT-PCR) reaction was then manipulated with particular primers (RIBOBIO, China) and SYBR Green PCR Master Mix (Thermo Fisher, USA) using the StepOnePlus Real-Time PCR System (Applied Biosystems). The reaction procedure is: denaturation at 95°C for 10 minutes, followed by 40 cycles of 95°C for 15 seconds, 60°C for 20 seconds, and 72°C for 12 seconds. The primers used in this investigation were: hsa_circ_0003220 forward: 5′-GAGGAGAGAACCCTATCCAGGG-3′ and reverse: 5′-CAGGTTAGATTTTAAAGTAGCCAACAAGACATC-3′; IGF1 forward: 5′-CGTCTCCCGTTCGCTAAATC-3′ and reverse: 5′-AATAAAAGCCCCGGTCTCCA-3′; miR-489-3p forward: 5′-GTCGTATCCAGTGCAGGGTCCGAGGTATTCGCACTGGATACGACGCTGCC-3′ and reverse: 5′-GCGCGTGACATCACATATAC-3′. glyceraldehyde 3-phosphate dehydrogenase forward: 5′-AGAAGGCTGGGGCTCATTTG-3′ and reverse: 5′-AGGGGCCATCCACAGTCTTC-3′; U6 forward: 5′-GGAACGATACAGAGAAGATTAGC-3′ and reverse: 5′-TGGAACGCTTCACGAATTTGCG-3′.

### 2.5. Cell Viability Assay

H460/PTX and A549/PTX cells were seeded (5000 cells/well) in 96-well plates after siRNA/plasmid transfection, and they were then subjected to DDP, docetaxel (DTX), and PTX after 48 hours. Then, cells were exposed to 2 mg/mL of 3-(4,5-dimethylthiazol-2-yl)-2,5-diphenyltetrazolium bromide (MTT) and let for 4 hours to react. After forming formazan crystals and dissolving them in 100 *μ*L of dimethylsulfoxide, the absorbance at 570 nm was determined. The PTX IC_50_ was calculated using the GraphPad Prism 7 (GraphPad Technologies, USA).

### 2.6. Dual-Luciferase Reporter Assay

The luciferase reporter plasmids hsa_circ_0003220-WT and IGF1-WT were created by cloning MiR-489-3p docking sites carrying hsa_circ_0003220 or IGF1 into psiCHECK2 (Promega). We also created the mutated plasmids (hsa_circ_0003220-MUT and IGF1-MUT) for the aforementioned luciferase reporter plasmids. The aforementioned vector was then co-incorporated into 293T with miR-489-3p or miR-NC, and the cells were then treated for 48 hours. The luciferase activity was measured using the dual-luciferase reporter (DLR) Assay Kit (Promega).

### 2.7. RNA Immunoprecipitation Assay

Millipore's EZ-Magna RIP RNA-Binding Protein Immunoprecipitation Kit (USA) was used for the RNA Immunoprecipitation (RIP) evaluation. H460/PTX and A549/PTX cells were lysed with Nase-I and RIP lysis buffer (Merck Millipore, USA), and 100 *μ*L of the lysate was then exposed to RIP buffer containing antibody-coated magnetic beads. hsa_circ_0003220 and miR-489-3p levels were then determined using RT-qPCR after being precipitated per the kit's instructions.

### 2.8. ELISA Assay

An enzyme linked immunosorbent assay (ELISA) kit (R&D Systems) was utilized in order to measure the amount of IGF1 present in the various culture media per the manufacturer's instructions. Every experiment was performed three times, and the results were analyzed and published based on the average.

### 2.9. Statistical Analysis

Triplicates of each experiment were run. Results were presented as mean ± standard deviation (SD). The one-way analysis of variance or Student's *t*-tests was used to analyze the difference. Spearman's correlation coefficient analysis was used to determine the relationship between miR-489-3p and either hsa_circ_0003220 or IGF1. The statistical significance threshold was set at *p* < 0.05.

## 3. Results

### 3.1. Hsa_circ_0003220 Expression in PR NSCLC Tissues and Cell Lines

**Figure 1:**
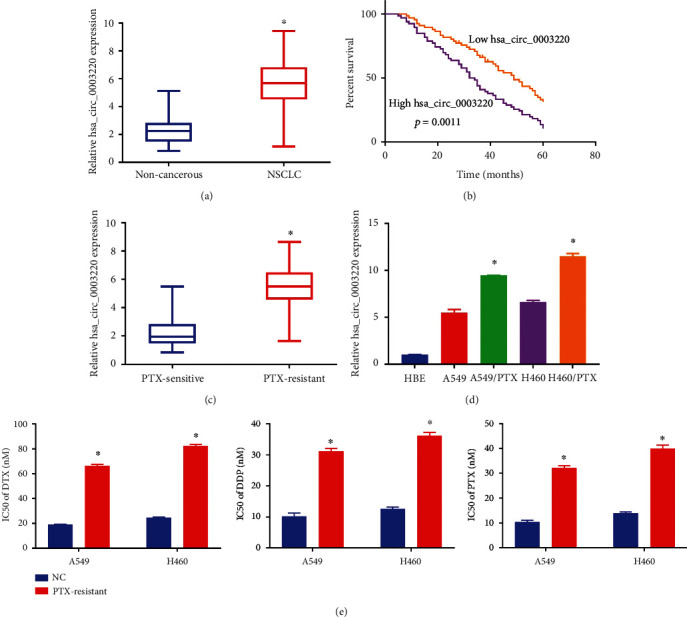
PR NSCLC cell lines and tissues exhibited robust expression of hsa_circ_0003220. (a) The expression of hsa_circ_0003220 in NSCLC samples is up-regulated compared with paracancerous tissue samples. (b) Survival assays of 132 NSCLC patients based on the hsa_circ_0003220 mean expression. (c) Hsa_circ_0003220 expression was analyzed by qRT-PCR in both PS and PR NSCLC samples. (d) qRT-PCR assay was employed to ascertain the hsa_circ_0003220 level in H460, A549, H460/PTX, and A549/PTX cells. (e) The MTT test was used to calculate the IC50 values of DTX, DDP, and PTX. ∗*p* < 0.05.

Through study on TMCO7, it was found that the expression levels of hsa_circ_0003220 in NSCLC specimens were noticeably greater than those in the matching normal specimens. The expression levels for hsa_circ_0003220 were evaluated and categorized as either low or high compared with the median value. We discovered that patients with high hsa_circ_0003220 expression had shorter overall survival than the hsa_circ_0003220-low group (Figure 1(b)). Consequently, there was a discernible upward increase in the level of hsa_circ_0003220 expression in PR NSCLC tissues compared with PS NSCLC tissues (Figure 1(c)). In addition, we discovered that the amount of hsa_circ_0003220 in H460 and A549 cells were lower than that in H460/PTX and A549/PTX cells and greater than that in HBE cells (Figure 1(d)). After that, we used the MTT test to determine whether H460/PTX and A549/PTX cells were resistant to PTX. Our findings showed increased these cells' IC50 values of DTX, DDP, and PTX (Figure 1(e)).

### 3.2. Suppression of Chemoresistance by hsa_circ_0003220 Knockdown in PR NSCLC Cells

**Figure 2:**
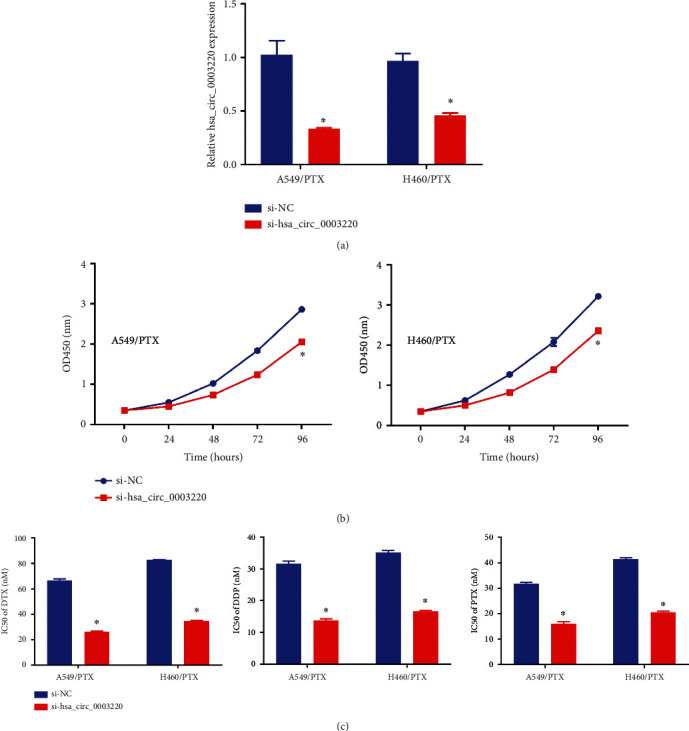
Hsa_circ_0003220 knockdown suppressed PTX resistance in PR NSCLC cells. (a) qRT-PCR assay was performed for hsa_circ_0003220 expression in H460/PTX and A549/PTX cells transfected with si-hsa_circ_0003220 or si-NC. (b) The MTT assay was used to determine cellular proliferation. (c) The MTT test was used to calculate the IC50 values of DTX, DDP, and PTX. ∗*p* < 0.05.

Loss-of-function experiments were performed by si-hsa_circ_0003220 transfection into H460/PTX and A549/PTX cells to inhibit the hsa_circ_0003220 expression. These experiments aimed to corroborate the involvement of hsa_circ_0003220 in the PTX resistance of NSCLC. In comparison with si-NC groups, the qRT-PCR test demonstrated that si-hsa_circ_0003220 transfection resulted in a striking drop in hsa_circ_0003220 expression in H460/PTX and A549/PTX cells (Figure 2(a)). MTT assay results showed that si-hsa_circ_0003220 transfection suppressed cellular proliferation in H460/PTX and A549/PTX cells (Figure 2(b)). Furthermore, these cells also had lower IC50 values for DTX, DDP, and PTX (Figure 2(c)), suggesting that si-hsa_circ_0003220 downregulation reduced the PTX resistance of these cells.

### 3.3. Hsa_circ_0003220 as the Sponge of miR-489-3p

**Figure 3:**
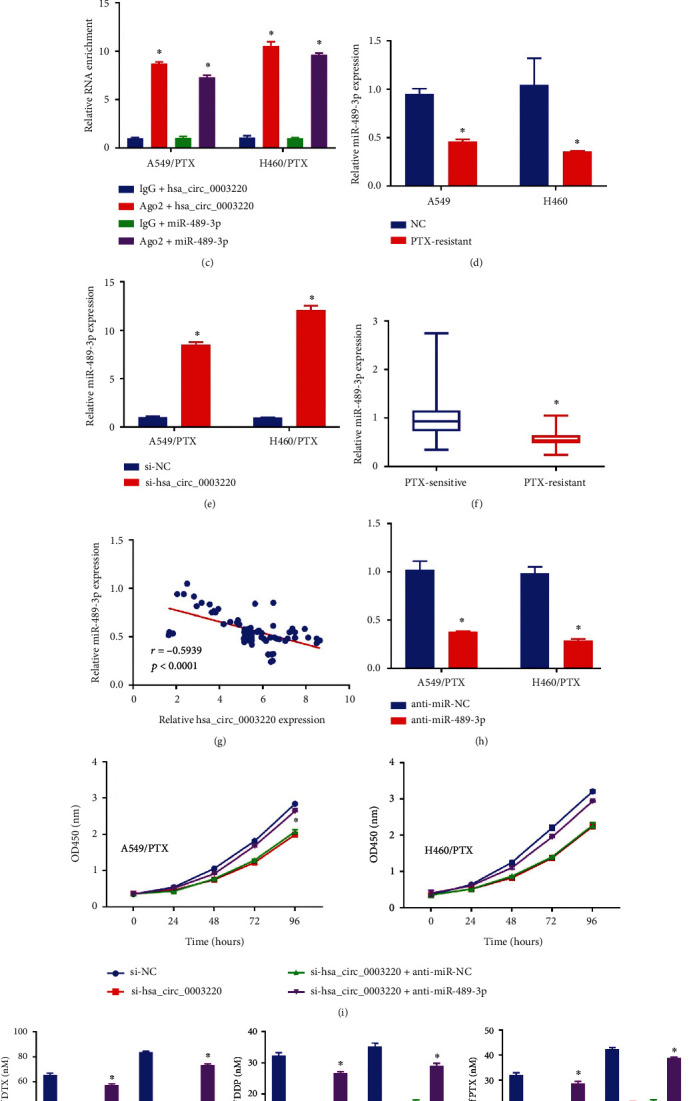
Hsa_circ_0003220 served as miR-489-3p's sponge. (a)The potential binding sites between hsa_circ_0003220 and miR-489-3p. (b) A DLR experiment to find out if hsa_circ_0003220 and miR-489-3p interacted in 293T cells. (c) RIP assay was performed to confirm whether hsa_circ_0003220 is bound to miR-489-3p. (d) qRT-PCR assay was employed to ascertain the miR-489-3p level in H460, A549, H460/PTX, and A549/PTX cells. (e) qRT-PCR analysis was employed to determine whether or not miR-489-3p expression was increased in H460/PTX and A549/PTX cells transfected with si-hsa_circ_0003220 or si-NC. (f) miR-489-3p expression was evaluated by qRT-PCR in both PS and PR NSCLC samples. (g) Spearman's correlation coefficient analysis estimated the correlation between miR-489-3p and hsa_circ_0003220 in PR tumor tissues. (h) miR-489-3p expression was quantified in anti-miR-NC and anti-miR-489-3p transfected H460/PTX and A549/PTX cells using a qRT-PCR technique. (i) The MTT assay was used to determine cellular proliferation. (j) The MTT test was used to calculate the IC50 values of DTX, DDP, and PTX. ∗*p* < 0.05.

We looked for putative miR docking sites within hsa_circ_0003220 to understand this protein's molecular pathway. MiR-489-3p's sequence was found to be complementary to hsa_circ_0003220 (Figure 3(a)). We performed a DLR test to support the hsa_circ_0003220-miR-489-3p connection further. Our results show that miR-489-3p significantly decreased the expression of the hsa_circ_0003220-WT luciferase gene (Figure 3(b)). RIP analysis revealed that the hsa_circ_0003220 and miR-489-3p genes were considerably enhanced in the Ago2 group in relation to the lgG group (Figure 3(c)). Additionally, compared with A549 and H460 cells, miR-489-3p levels in H460/PTX and A549/PTX were considerably lower (Figure 3(d)). Depletion of hsa_circ_0003220 also increased miR-489-3p levels (Figure 3(e)). Additionally, miR-489-3p levels in PR tissues were significantly lower than in their sensitive counterparts (Figure 3(f)). Furthermore, the miR-489-3p levels were inversely correlated with those of hsa_circ_0003220 (Figure 3(g)). We showed a significant decrease in miR-489-3p levels upon the inclusion of anti-miR-489-3p (Figure 3(h)). Finally, we showed that suppressing hsa_circ_0003220 inhibited cell proliferation (Figure 3(i)) and DTX, DDP, and PTX IC_50_ (Figure 3(j)). However, anti-miR-489-3p insertion into H460/PTX and A549/PTX cells restored the low IC50 values. By explicitly targeting miR-489-3p, hsa_circ_0003220 as a whole negatively impacted miR-489-3p expression.

### 3.4. IGF1 Was a Direct Target Gene of miR-489-3p

**Figure 4:**
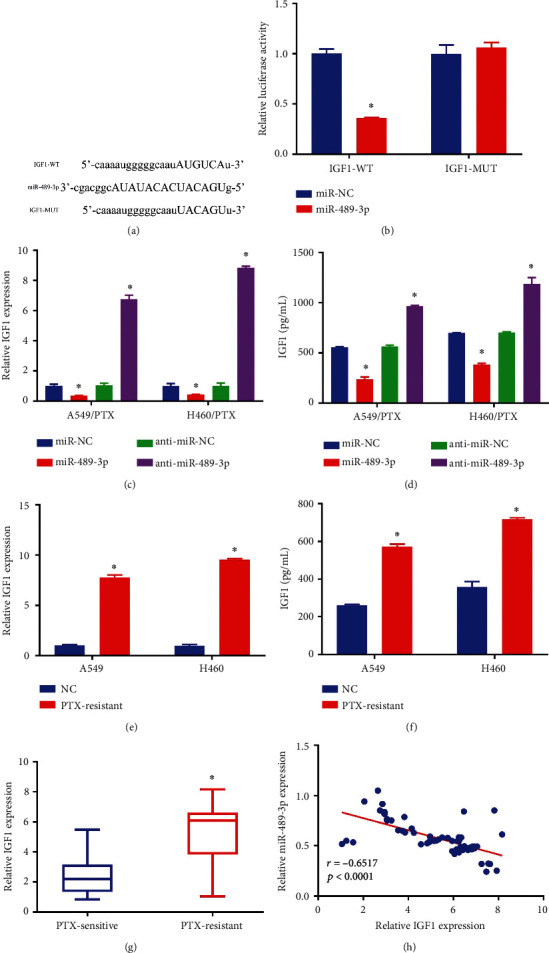
IGF1, a direct miR-489-3p target gene. (a) MiR-489-3p and IGF1 3′-untranslated region (UTR) binding sites, predicted computationally. (b) DLR assay to examine the cooperation between IGF1 and miR-489-3p in 293T cells. (c–f) A combination of qRT-PCR and ELISA was used to quantify IGF1 expression. (g) IGF1 levels in PR and PS NSCLC tissues were analyzed using qRT-PCR. (h) Spearman's correlation coefficient analysis estimated the correlation between IGF1 and miR-489-3p in PR tumor tissues. ∗*p* < 0.05.

StarBase 3.0 analysis revealed that miR-489-3p targets the gene IGF1, and Figure 4(a) illustrates their putative binding sites. A DLR experiment then verified the connection between miR-489-3p and IGF1. The findings demonstrated that, in contrast to miR-NC and IGF1-WT co-transfected groups, luciferase activity was inhibited in miR-489-3p and IGF1-wt co-transfected 293T cells. Still, no difference was seen in IGF1-MUT groups (Figure 4(b)). Then, to investigate how miR-489-3p affects the expression of IGF1, we transfected miR-489-3p, anti-miR-489-3p, or respective controls. MiR-489-3p and anti-miR-489-3p were transfected into H460/PTX and A549/PTX cells, as shown in Figures 4(c) and 4(d). We discovered that anti-miR-489-3p had the opposite effects from those of miR-489-3p overexpression in terms of reducing the IGF1 expression in H460/PTX and A549/PTX cells. Additionally, our findings demonstrated that A549 and H460 cells expressed less IGF1 than H460/PTX and A549/PTX cells (Figures 4(e) and 4(f)). PR tumor tissues showed increased IGF1 expression compared with PS tumor tissues (Figure 4(g)). In PR NSCLC tissues, the level of IGF1 mRNA was discovered to be inversely linked with miR-489-3p expression using Spearman's correlation coefficient analysis (Figure 4(h)). In conclusion, miR-489-3p directly influenced IGF1 expression negatively.

### 3.5. Repression of Cell Progression and Enhancement of PTX Sensitivity by miR-489-3p Overexpression Targeting IGF1

**Figure 5:**
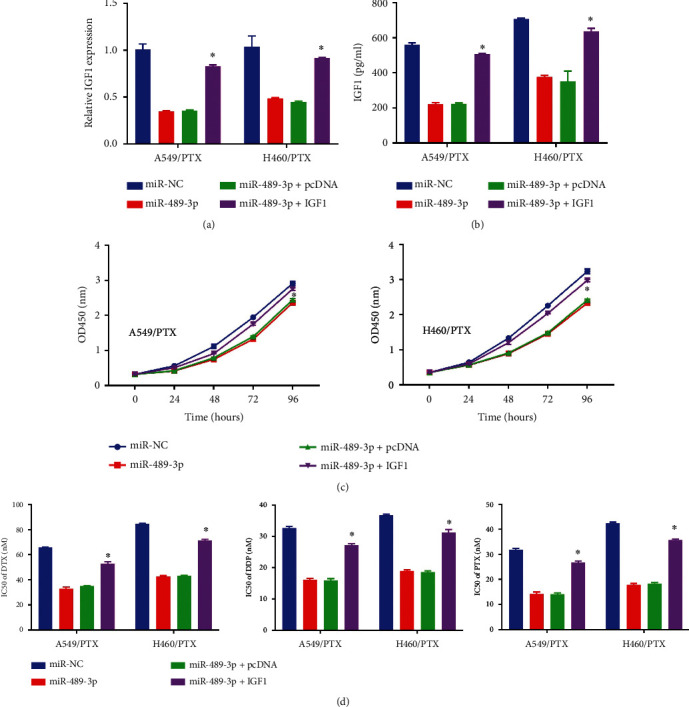
In PR NSCLC cells, overexpression of miR-489-3p increased sensitivity to PTX and suppressed cell growth via inhibiting IGF1. (a and b) A combination of qRT-PCR and ELISA was used to quantify IGF1 expression. (c) The MTT assay was used to determine cellular proliferation. (d) The MTT test was used to calculate the IC50 values of DTX, DDP, and PTX. ∗*p* < 0.05.

miR-489-3p, miR-NC, miR-489-3p + IGF1, or miR-489-3p + pcDNA transfected, H460/PTX, and A549/PTX were used to assess the involvement of miR-489-3p and IGF1 in PTX resistance. MiR-489-3p transfection lowered the IGF1 expression in H460/PTX and A549/PTX cells, but IGF1 transfection countered these effects (Figures 5(a) and 5(b)). Through functional tests, we discovered that the overexpression of miR-489-3p in the A549/PTX and H460/PTX cells significantly lowered PTX resistance (Figure 5(c)) and reduced DTX, DDP, and PTX IC50 (Figure 5(d)). At the same time, the increase in IGF1 successfully overrode the effects.

### 3.6. Decreased IGF1 Expression through miR-489-3p Sponging by hsa_circ_0003220 Knockdown

**Figure 6:**
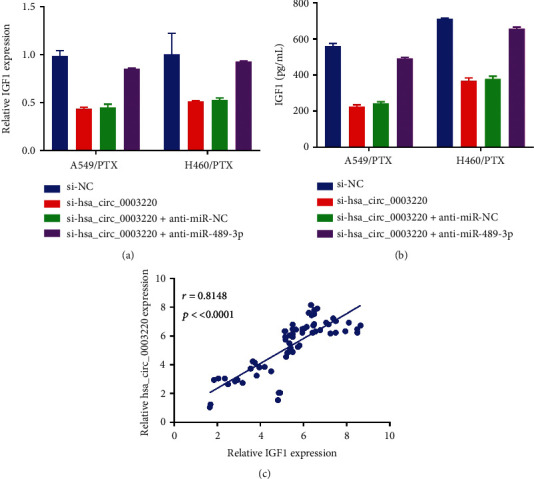
Reducing IGF1 expression by sponging miR-489-3p, achieved by knocking down hsa_circ_0003220. (a and b) A combination of qRT-PCR and ELISA was used to quantify IGF1 expression. (c) Spearman's correlation coefficient analysis estimated the correlation between IGF1 and hsa_circ_0003220 in PR tumor tissues. ∗*p* < 0.05.

To examine the relationships between hsa_circ_0003220, miR-489-3p, and IGF1, H460/PTX and A549/PTX cells' transfection was performed with si-NC, si-hsa_circ_0003220, si-hsa_circ_0003220 + anti-miR-NC, or si-hsa_circ_0003220 + anti-miR-489-3p. As seen in Figures 6(a) and 6(b), anti-miR-489-3p successfully restored the silencing effects of hsa_circ_0003220, significantly reducing the IGF1 expression in H460/PTX and A549/PTX cells. In PR NSCLC tissues, hsa_circ_0003220 was discovered to have a positive connection with IGF1 expression using Spearman's correlation coefficient analysis (Figure 6(c)). Thus, we deduced that hsa_circ_0003220 favourably regulated IGF1 expression in PR NSCLC cells through miR-489-3p sponging.

## 4. Discussion

Chemotherapy resistance is a significant obstacle to treating human malignancies, particularly NSCLC. The advancement of technology has made it possible to confirm the involvement of a variety of circRNAs in controlling the growth of chemoresistance. This study aimed to investigate the functions that hsa_circ_0003220 plays in modulating the chemoresistance of NSCLC. Consequently, chemoresistance was made easier in PR NSCLC cells by hsa_circ_0003220, which regulated the miR-489-3p/IGF1 axis.

Researchers have slowly but surely begun to focus more on the critical functions that circRNAs play in the evolution of NSCLC tumors and the development of treatment resistance. For instance, circ_0011292 improves PTX resistance in NSCLC via modifying the miR-379-5p/TRIM65 axis [18]. By the miR-433-3p/CHEK1 axis regulation, the knockdown of circ_0011292 in PR NSCLC cells slows the course of the disease and reduces treatment resistance [19]. By miR-512-5p sponging to modify FAM83F expression, blocking circ_0010235, which is responsible for acquired PTX resistance in NSCLC, may help to reduce it [20]. Based on these findings, circRNAs seemed to play many roles in developing treatment resistance in NSCLC. Concerning hsa_circ_0003220, it was discovered that chemoresistant NSCLC tissues and cells included an elevated amount of this particular circRNA. In PR NSCLC cells, a lack of hsa_circ_0003220 inhibited both the cells' proliferation and their resistance to PTX in vitro. Hsa_circ_0003220 was shown to contribute to NSCLC's cell proliferation and chemoresistance collectively.

After examining the process, it was found that hsa_circ_0003220 served as a sponge for miR-489-3p, increasing the production of IGF1. CircCDK1 knockdown reduces CDK1 expression by targeting miR-489-3p to suppress the development of breast cancer and strengthen the sensitivity of tamoxifen [21]. Expression of mir-489-3p acts as an effective marker of poor prognosis in patients with NSCLC [22]. MiR-489-3p inhibits proliferation and migration of bladder cancer cells through downregulation of histone deacetylase 2 [23]. All of these data pointed to the possibility that miR-489-3p played a vital role in slowing down NSCLC. In this study, the findings demonstrated that PR NSCLC cells and tissues had lower levels of the microRNA known as miR-489-3p. In PR NSCLC cells, inhibiting miR-489-3p may successfully restore the effects that hsa_circ_0003220 knockdown had on cell proliferation and PTX sensitivity. In addition, miR-489-3p overexpression decreased cell proliferation and increased susceptibility to PTX in PR cells; however, these effects may be eliminated by increasing IGF1 levels. Based on our findings, miR-489-3p was able to target IGF1 and inhibit cell proliferation in PR NSCLC cells. This led to a reduction in PTX resistance in these cells. Downregulation of lncRNA H19, which regulates the miR-18b/IGF1 axis, is said to make melanoma cells more sensitive to the effects of DDP [24], which is in agreement with our findings. Through the miR-379-5p/IGF1 axis, inhibiting hsa_circ_0074027 reduced chemoresistance in NSCLC [25]. IGF1 has already been found to be a target of numerous miRNAs, including miR-186-3p [26], miR-1-3p [27], and miR-4500 [28]. However, this is the first study to identify the role of miR-489-3p and IGF1 in the chemoresistance of NSCLC. However, there are still significant restrictions on what can be concluded from this research. For instance, the sample size was insufficient, and we did not conduct in-depth research on the roles that IGF1 plays in developing chemoresistance in NSCLC.

In conclusion, our research showed that hsa_circ_0003220 increased PTX resistance in NSCLC via modifying the miR-489-3p/IGF1 axis. This might potentially give new strategies to overcome the chemoresistance seen in NSCLC.

## Data Availability

The data used to support the findings of this study are available from the corresponding author upon request.
